# ECP versus ruxolitinib in steroid-refractory chronic GVHD – a retrospective study by the EBMT transplant complications working party

**DOI:** 10.1038/s41409-023-02174-2

**Published:** 2024-01-06

**Authors:** Olaf Penack, Christophe Peczynski, William Boreland, Jessica Lemaitre, H. Christian Reinhardt, Ksenia Afanasyeva, Daniele Avenoso, Tobias A. W. Holderried, Brian Thomas Kornblit, Eleni Gavriilaki, Carmen Martinez, Patrizia Chiusolo, Maria Caterina Mico, Elisabeth Daguenet, Stina Wichert, Hakan Ozdogu, Agnieszka Piekarska, Francesca Kinsella, Grzegorz W. Basak, Hélène Schoemans, Christian Koenecke, Ivan Moiseev, Zinaida Peric

**Affiliations:** 1 Medical Clinic, Department for Haematology, Oncology and Tumorimmunology, Charité Universitätsmedizin Berlin, Berlin, Germany; 2 EBMT Transplant Complications Working Party, Paris, France; 3 CHU de Saint-Etienne, Hematology, Saint-Etienne, France; 4 University Hospital, Essen, Germany; 5 RM Gorbacheva Research Institute, Pavlov University, St Petersburg, Russia; 6 Kings’ College Hospital, London, UK; 7 Department of Oncology, Hematology, Immuno-Oncology and Rheumatology, University Hospital Bonn, Bonn, Germany; 8 Rigshospitalet, Copenhagen, Denmark; 9 Papanicolaou G. Hospital, Thessaloniki, Greece; 10 Hematopoietic Stem Cell Unit, Hematology Department, ICMHO, Hospital Clínic of Barcelona, University of Barcelona, Barcelona, Spain; 11 Fondazione Policlinico Universitario A. Gemelli IRCCS-Università Cattolica del Sacro Cuore-Roma, Roma, Italy; 12 ASST Papa Giovanni XXIII, Bergamo, Italy; 13 Institut de Cancerologie Lucien Neuwirth, Saint-Étienne, France; 14 Skåne University Hospital in Lund, Lund, Sweden; 15 Department of Hematology, Baskent University Hospital, Adana, Türkiye; 16 Department of Hematology and Transplantology, University Clinical Center and Medical University of Gdansk, Gdańsk, Poland; 17 University Hospital Birmingham NHS Trust, Birmingham, UK; 18 Department of Hematology, Oncology and Internal Medicine, the Medical University of Warsaw, Warsaw, Poland; 19 Department of Hematology, University Hospitals Leuven, Leuven, Belgium; 20 Department of Public Health and Primary Care, ACCENT VV, KU Leuven - University of Leuven, Leuven, Belgium; 21 Department of Hematology, Hemostasis, Oncology and Stem Cell Transplantation, Hannover Medical School, Hannover, Germany; 22 Department of Haematology, University Hospital Centre Rijeka, Rijeka, Croatia

**Keywords:** Translational research, Stem-cell research

## Abstract

Ruxolitinib has become the new standard of care for steroid-refractory and steroid-dependent chronic GVHD (SR-cGVHD). Our aim was to collect comparative data between ruxolitinib and extracorporeal photophoresis (ECP). We asked EBMT centers if they were willing to provide detailed information on GVHD grading, -therapy, -dosing, -response and complications for each included patient. 31 centers responded positively and we included all patients between 1/2017-7/2019 treated with ECP or ruxolitinib for moderate or severe SR-cGVHD. We identified 84 and 57 patients with ECP and ruxolitinib, respectively. We performed multivariate analyses adjusted on grading and type of SR-cGVHD (steroid dependent vs. refractory vs. intolerant to steroids). At day+180 after initiation of treatment for SR-cGVHD the odds ratio in the ruxolitinib group to achieve overall response vs. the ECP group was 1.35 (95% CI = [0.64; 2.91], *p* = 0.43). In line, we detected no statistically significant differences in overall survival, progression-free survival, non-relapse mortality and relapse incidence. The clinical significance is limited by the retrospective study design and the current data can’t replace prospective studies on ECP in SR-cGVHD. However, the present results contribute to the accumulating evidence on ECP as an effective treatment option in SR-cGVHD.

## Background

The use of allogeneic stem cell transplantation (alloSCT) is constantly increasing with nearly 20.000 transplantations reported to the European Society for Blood and Marrow Transplantation (EBMT) per year [1]. Chronic GVHD (cGVHD) remains one of the major concerns causing considerable morbidity and mortality. In a recent CIBMTR analysis, the incidence of cGVHD was 54% among recipients of matched-related donor (MRD) grafts and 53% among recipients of matched-unrelated donor (MUD) grafts, again highlighting that cGVHD is a frequent event after alloSCT [2]. In the same manuscript, a highly significant increase in non-relapse mortality (NRM) of alloSCT recipients with cGVHD vs. control alloSCT recipients was described. Hazard ratios depend on age but were in a range between 1.4-2.0 [2], showing that cGVHD not only impacts the quality of life but also is a robust risk factor of mortality.

Moderate to severe forms of cGVHD are usually treated with steroids, such as 1 mg/Kg body weight of Prednisolone [3]. In case the treatment with steroids is not successful, the term steroid-refractory cGVHD (SR-cGVHD) is used. There is no published high-quality data from larger multicenter patient populations on the incidence of SR-cGVHD.

The treatment with ruxolitinib has evolved as a new therapeutic standard for patients with SR-cGVHD [4]. However, patients with SR-cGVHD have typicallly a high mortality despite effective novel drugs, such as ruxolitinib, and there is urgent medical need to improve treatment strategies.

Extracorporal photopheresis (ECP) includes ultraviolet A light irradiation and 8methoxypsoralen exposure of autologous peripheral blood monunuclear cells. ECP has been successfully used for treatment of SR-cGVHD as an alternative or an add-on to standard immunosuppression [5–11]. A favorable safety profile regarding infectious disease risk as well as a steroid sparing effect, when combined with steroid treatment, have been suggested as potential advantages of ECP [9].

Due to the absence of a general availability of ECP and also reflecting the lack of randomized trials comparing ECPs efficacy and toxicity with newer treatment options including ruxolitinib, there is a high variety between treatment centers regarding their use of ECP. While studies have shown the effectiveness and safety of ECP in SR-cGVHD treatment, there is limited data to show how it is being used in the real world setting since ruxolitinib became available.

In the current study we used the EBMT database to retrospectively study treatment patterns and outcomes of SR-cGVHD treatment with ECP versus ruxolitinib. Our aim was to improve the evidence basis on the potential benefit of ECP use as treatment of SR-cGVHD in the current treatment landscape.

## Methods

This is a retrospective multicentre analysis using the data set of the EBMT registry. The EBMT is a voluntary working group of more than 600 transplant centres that are required to report regular follow up on all consecutive stem cell transplantations. Audits are routinely performed to determine the accuracy of the data. The study was planned and approved by the Transplant Complications Working Party of the EBMT. All patients gave their written informed consent to use their personal information for research purposes. The study was conducted in accordance with the Declaration of Helsinki and Good Clinical Practice guidelines.

We identified 227 EBMT centers that use ECP and asked them if they were willing to participate in this study by completing a data form (Med-C, supplementary data) with very detailed information on GVHD grading, -therapy, -dosing, -response and complications for each included patient. 31 centers responded positively (14%) and we included all patients receiving alloSCT between 1/2017-7/2019 and treated with ECP or Ruxolitinib for moderate or severe SR-cGVHD from these centers.

### Inclusion criteria

- Patients who develop SR-cGVHD after first alloSCT on/or after Jan 1st 2017 but before Jan 1st 2019
- Patients who initiated treatment with ECP or Ruxolitinib within 1 year of the onset of SR-cGvHD
- Severity: moderate to severe only at time of treatment initiation
- Patients who are ≥ 18 years at time of treatment initiation

### Exclusion criteria

- Patients on a clinical trial for GVHD in the retrospective period
- Patient is pregnant or breastfeeding
- Patients who received ECP or ruxolitinib before the onset of steroid-refractory acute GVHD

Data collected included recipient and donor characteristics (age, sex, cytomegalovirus serostatus and Karnofsky performance status score), diagnosis and status at transplant, interval from diagnosis to transplant, and transplant-related factors, including conditioning regimen, use of anti-thymocyte globulin or Alemtuzumab for pre-transplant in vivo T- cell depletion, stem cell source, ex-vivo T-cell depletion and post-transplant GVHD prophylaxis. Grading of cGVHD was performed using established criteria [12, 13]. For the purpose of this study, all necessary data were collected according to the EBMT guidelines, using the EBMT Minimum Essential Data forms as well as Med-C forms (see supplementary data).

### Statistical analysis

The primary endpoint was overall response rate (ORR) at 180 days after initiation of treatment. Secondary endpoints comprised classical survival outcomes: Overall Survival (OS), Progression-Free Survival (PFS), Relapse Incidence (RI) and Non-Relapse-Mortality (NRM), as well as incidence of infectious complications. Start time was the date of start of ECP or ruxolitinib for all endpoints.

ORR at 180 days was defined as being in complete or partial response to the treatment 180 days after introduction of treatment. Death before 180 days was considered as a failure of the treatment. NRM was defined as death without relapse/progression and PFS was defined as survival without relapse or progression.

Multivariate logistic regression models were used to evaluate ORR and results were given as odd ratios. OS and PFS were calculated using the Kaplan-Meier method. Cumulative incidence functions were used to estimate RI and NRM in a competing risk setting, death and relapse competing with each other [13]. For the estimation of the cumulative incidence of infectious complications, relapse and death were considered to be competing events. Multivariate analyses were performed using the Cox proportional-hazards model for all survival endpoints. All tests were 2-sided. Statistical analyses were performed with R 4.1.2 software (R Development Core Team, Vienna, Austria) packages.

## Results

### Patient- and transplantation characteristics

We identified 84 and 57 patients with moderate or severe SR-cGVHD who were treated with ECP or ruxolitinib, respectively between January 1st, 2017 and July 1st, 2019 in the EBMT database. Major patient- disease- and transplant characteristics were evenly distributed between the groups (Table 1). Patients were mainly transplanted for Acute Leukemia (52.5%), MDS/MPN (31.2%), Lymphoma (8.5%), Chronic Leukemia (4.3%). Stem cell donors were mainly unrelated (53.2%), identical siblings (40%) or haploidentical (6.4%). Patient median age was 55.1 years, with a majority of male recipients (57.4%) and male donors (54.6%). In vivo T-cell depletion with anti-T-cell globulin (ATG, also termed anti-thymocyte globulin) or Campath was given in 48.6%. GVHD prophylaxis was mainly calcineurin inhibitor + methotrexate in 50.4 %, calcineurin inhibitor + mycophenolate mofetil in 27% and post transplantation cyclophosphamide based in 14.9%.

**Table 1:** Characteristics of both cohorts.

Variable	Level	ECP (n = 84)	Ruxo(n = 57)	Overall (n = 141)	P-value
Year of transplantation	median (min-max) [IQR]	2017 (2014-2019) [2016-2018]	2017 (2016-2019) [2017-2018]	2017 (2014-2019) [2016-2018]	0.54
Cell source	Bone marrow	6 (7.1%)	6 (10.5%)	12 (8.5%)	Not done
	Peripheral blood	76 (90.5%)	51 (89.5%)	127 (90.1%)	
	Cord blood	2 (2.4%)	0 (0%)	2 (1.4%)	
Type of donor	Matched related	32 (38.6%)	24 (42.1%)	56 (40%)	Not done
	MUD 10/10	31 (37.3%)	22 (38.6%)	53 (37.9%)	
	mMUD 9/10	4 (4.8%)	8 (14%)	12 (8.6%)	
	mMUD 8/10 or less	2 (2.4%)	0 (0%)	2 (1.4%)	
	UD (unknown mismatch)	8 (9.6%)	0 (0%)	8 (5.7%)	
	Haploidentical	6 (7.2%)	3 (5.3%)	9 (6.4%)	
Diagnosis	Acute leukaemia	41 (48.8%)	33 (57.9%)	74 (52.5%)	Not done
	Chronic leukaemia	3 (3.6%)	3 (5.3%)	6 (4.3%)	
	Lymphoma	7 (8.3%)	5 (8.8%)	12 (8.5%)	
	Myelodysplastic/ Myeloproliferative	29 (34.5%)	15 (26.3%)	44 (31.2%)	
	Non-malignant	4 (5%)	1 (1.8%)	5 (3.5%)	
Complete remission at transplant	CR	40 (50%)	34 (60.7%)	74 (54.4%)	0.22
	No CR	40 (50%)	22 (39.3%)	62 (45.6%)	
	missing	4	1	5	
Patient age (years)	median (min-max) [IQR]	55.5 (20.6-77.9) [42.3-63.3]	52.7 (17.7-71.3) [39.2-60.1]	55.1 (17.7-77.9) [40.1-62.1]	0.40
Patient sex	Male	48 (57.1%)	33 (57.9%)	81 (57.4%)	0.93
	Female	36 (42.9%)	24 (42.1%)	60 (42.6%)	
Karnofsky score	>= 90	36 (67.9%)	30 (75%)	66 (71%)	0.12
	< 90	17 (32.1%)	10 (25%)	27 (29%)	
	Missing	60 (73.2%)	48 (84.2%)	108 (77.7%)	
HCT-CI Sorror score	0	40 (49.4%)	24 (43.6%)	64 (47.1%)	0.063
	1 or 2	28 (34.6%)	13 (23.6%)	41 (30.1%)	
	3+	13 (16%)	18 (32.7%)	31 (22.8%)	
	missing	3	2	5	
Intensity of conditioning	RIC	41 (49.4%)	22 (39.3%)	63 (45.3%)	0.24
	MAC	42 (50.6%)	34 (60.7%)	76 (54.7%)	
	missing	1	1	2	
TBI	No	68 (81%)	43 (75.4%)	111 (78.7%)	0.43
	Yes	16 (19%)	14 (24.6%)	30 (21.3%)	
In vivo T-cell depletion	ATG / Campath	47 (56%)	21 (37.5%)	68 (48.6%)	0.032
	No	37 (44%)	35 (62.5%)	72 (51.4%)	
	missing	0	1	1	
Type of GVHD prophylaxis	CNI + MTX	41 (48.8%)	30 (52.6%)	71 (50.4%)	
	CNI + MMF	27 (32.1%)	10 (17,5%)	38 (27%)	
	PTCY based	10 (11.9%)	11 (19.3%)	21 (14.9%)	
	Other	6 (7.1%)	6 (10.5%)	12 (8.5%)	
	Missing	0	1	1	

*ATG* anti-T-cell globulin, *CNI* calcineurin inhibitors, *HCT-CI* hematopoietic cell transplantation comorbidity index, *MMF* mycophenolate mofetil, *MUD* matched unrelated donor, *MTX* methotrexate, *TBI* total body irradiation.

The median follow up time was 33.6 months [95% CI 30.8–38.1 months] in the ECP group and 24.7 months [95% CI 22.3–28.9] in the ruxolitinib group.

### Characteristics of SR-cGVHD

SR-cGVHD is described in Table 2. The majority of patients (68.1%) had been treated with additional drugs/strategies for SR-cGVHD before ECP or ruxolitinib was started. These included most frequently calcineurin inhibitors and mycophenolate mofetil, but also etanercept, mesenchymal stroma cells, methotrexate, vedolizumab, imatinib and low-dose interleukin-2 have been used.

**Table 2:** Characteristics of chronic GVHD.

Variable	Level	ECP (n = 84)	Ruxo(n = 57)	Overall (n = 141)	P-value
Type of steroid	Prednisone	69 (82.1%)	42 (73.7%)	111 (78.7%)	Not done
	Methylprednisone	15 (17.9%)	15 (26.3%)	30 (21.3%)	
Steroid initial dose (mg/kg/day)	median (min-max) [IQR]	1 (0.1-80) [0.5-1]	1 (0.1-2) [0.4-1]	1 (0.1-80) [0.5-1]	Not done
	missing	1	2	3	
Time between start and end of steroids (days)	median (min-max) [IQR]	239 (3-1028) [93.8-474.8]	196 (3-1709) [95.2-391.8]	218.5 (3-1709) [93.8-411.2]	Not done
	missing	22	17	39	
Other systemic drugs or strategies used to treat aGvHD (other than steroids)	No other drugs / strategies	27 (32.1%)	18 (31.6%)	45 (31.9%)	Not done
	CNI	32 (38.1%)	25 (43.9%)	57 (40.4%	
	MMF	18 (21.4%)	14 (24.6%)	32 (22.7%)	
	Others #	10 (11.9%)	8 (14%)	18 (12.7%)	
Time start steroids to SR onset (days)	median (min-max) [IQR]	78 (0-543) [23-196.8]	126 (0-1037) [41-370.2]	94 (0-1037) [28-237]	Not done
Type of steroid refractory	Steroid-dependant	34 (40.5%)	13 (22.8%)	47 (33.3%)	0.038
	Steroid-intolerant	13 (15.5%)	17 (29.8%)	30 (21.3%)	
	Steroid-refractory	37 (44%)	27 (47.4%)	64 (45.4%)	
Chronic GVHD overall grade (at start of SR treatment)	Moderate	30 (35.7%)	21 (36.8%)	51 (36.2%)	
	Severe	54 (64.3%)	36 (63.2%)	90 (63.8%)	0.89
Skin NIH score (at start of SR treatment)	0	18 (22%)	16 (29.1%)	34 (24.8%)	
	1	19 (23.2%)	7 (12.7%)	26 (19%)	Not done
	2	21 (25.6%)	12 (21.8%)	33 (24.1%)	
	3	24 (29.3%)	20 (36.4%)	44 (32.1%)	
	missing	2	2	4	
Liver NIH score (at start of SR treatment)	0	53 (65.4%)	36 (64.3%)	89 (65%)	
	1	11 (13.6%)	11 (19.6%)	22 (16.1%)	Not done
	2	8 (9.9%)	6 (10.7%)	14 (10.2%)	
	3	9 (11.1%)	3 (5.4%)	12 (8.8%)	
	missing	3	1	4	
Lower GI NIH score (at start of SR treatment)	0	61 (77.2%)	41 (73.2%)	102 (75.6%)	
	1	4 (5.1%)	6 (10.7%)	10 (7.4%)	
	2	6 (7.6%)	3 (5.4%)	9 (6.7%)	Not done
	3	8 (10.1%)	6 (10.7%)	14 (10.4%)	
	missing	5	1	6	
Upper GI NIH score (at start of SR treatment)	0	70 (88.6%)	47 (85.5%)	117 (87.3%)	
	1	3 (3.8%)	6 (10.9%)	9 (6.7%)	Not done
	2	3 (3.8%)	1 (1.8%)	4 (3%)	
	3	3 (3.8%)	1 (1.8%)	4 (3%)	
	missing	5	2	7	
Mouth NIH score (at start of SR treatment)	0	33 (41.2%)	13 (23.2%)	46 (33.8%)	
	1	25 (31.2%)	21 (37.5%)	46 (33.8%)	Not done
	2	17 (21.2%)	15 (26.8%)	32 (23.5%)	
	3	5 (6.2%)	6 (10.7%)	11 (8.1%)	
	missing	4	2	6	
Eyes NIH score (at start of SR treatment)	0	49 (63.6%)	28 (50.9%)	77 (58.3%)	
	1	14 (18.2%)	10 (18.2%)	24 (18.2%)	
	2	12 (15.6%)	9 (16.4%)	21 (15.9%)	Not done
	3	2 (2.6%)	7 (12.7%)	9 (6.8%)	
	missing	7	2	9	
Lung NIH score (at start of SR treatment)	0	61 (79.2%)	43 (78.2%)	104 (78.8%)	
	1	5 (6.5%)	4 (7.3%)	9 (6.8%)	
	2	8 (10.4%)	5 (9.1%)	13 (9.8%)	Not done
	3	3 (3.9%)	3 (5.5%)	6 (4.5%)	
	missing	7	2	9	

*CNI* calcineurin inhibitors, *MMF* mycophenolate mofetil.

# Others: Etanercept, mesenchymal stroma cells, methotrexate, vedolizumab, imatinib, interleukin-2.

Of note, when we investigated the type of cGHVD, we found that more patients in the ECP group had steroid-dependant cGVHD vs. the ruxolitinib group (40.5% vs. 22.8%, *p* = 0.038). On the other hand the ruxolitinib group contained more patients suffering from steroid-intolerance (29.8%) vs. the ECP group (15.5%). The severity of cGVHD at start of treatment was not statistically different between the two groups (64.3% severe in the ECP group, 63.2% in the ruxolitinib group, *p* = 0.89).

### Key efficacy outcome parameters

The primary outcome in our study was overall response rate (ORR) at day +180 after initiation of ECP or Ruxolitinib. In the ECP group ORR at +180 days was 45.7% (95% CI = [34.6; 57.1]) vs. 56.1% (95% CI = [42.3; 69.3]) in the ruxolitinib group.

We next performed multivariate analysis adjusted on the type of SR-cGVHD (ref: refractory vs. dependent vs. intolerant), cGVHD grade (ref: moderate vs. severe) and the hematopoietic cell transplantation comorbidity index (HCT-CI, Sorror Score, ref: 0 vs. 1-2 vs. 3 + ). We found no statistically significant differences in ORR at day +180 between ECP and ruxolitinib (Table 3). The odd ratio in the ruxolitinib group to achieve overall response vs. the ECP group was 1.35 (95% CI = [0.64; 2.91], *p* = 0.43). As expected, severe chronic GVHD was a significant risk factors for not achieving an overall response at day +180 (OR = 0.39, 95% CI = [0.18; 0.83], *p* = 0.016). In addition an HCT-CI of 1-2 vs. 0 was significantly associated with not achieving an overall response (OR = 0.36, 95% CI = [0.15; 0.83], *p* = 0.02). In contrast steroid-refractory vs. steroid-dependent vs. steroid-intolerant cGVHD had no significant association with achieving an overall response.

**Table 3:** Multivarate analyses. Results are given for the ruxolitinib group with the ECP group being the reference.

Variable	Hazard ratio/Odd ratio [95% CI]	*P*
Overall response rate at day +180	1.35 [0.64;2.91]	0.43
Overall survival	0.71 [0.32–1.6]	0.41
Progression-free survival	0.74 [0.4–1.36]	0.33
Relapse incidence	0.61 [0.17–2.15]	0.44
Non-relapse mortality	0.72 [0.32–1.63]	0.43

**Fig. 1:**
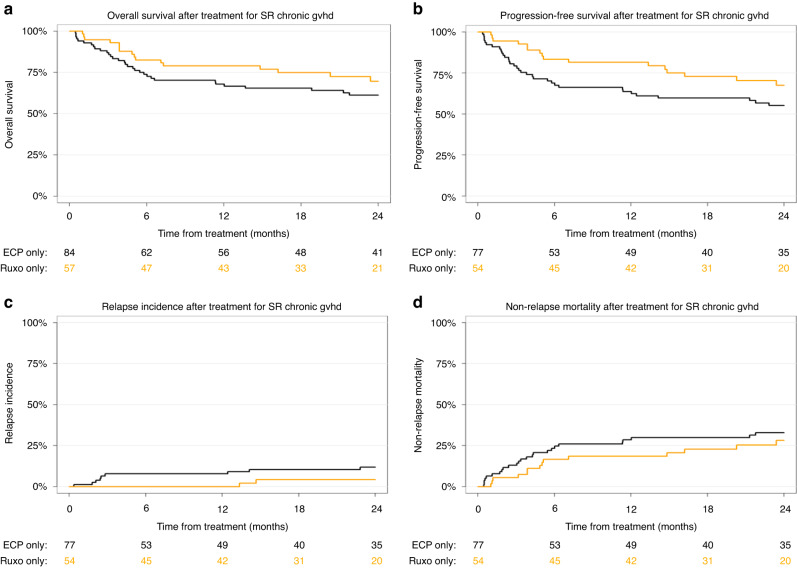
Univariate outcome graphs. Shown are overall suvival (**a**), progression-free survival (**b**), relapse incidence (**c**) and non-relapse mortality (**d**) in patients with SR-cGVHD after initiation of treatment with ECP (black lines – –) or Ruxolitinib (orange lines).

We detected no statistically significant differences in survival or relapse of the underlying malignancy between the two ECP and ruxolitinib SR-cGVHD cohorts. Univariate outcome graphs are shown in Fig. 1: overall survival (Fig. 1a), progression free survival (Fig. 1b), relapse incidence (Fig. 1c) and non-relapse mortality (Fig. 1d). The results of the multivariate analyses are given in Table 3.

Hazard ratios for the ruxolitinib group with the ECP group being the reference were for overall survival 0.71 [95% CI 0.32–1.60], progression free survival 0.74 [95% CI 0.40–1.36], relapse incidence 0.61 [95% CI 0.17–2.15] and non-relapse mortality 0.72 [95% CI 0.32–1.63].

### Safety-infectious complications

Infections occurred frequently in these high-risk patients with SR-cGVHD. The most common were bacteraemia and viremia with a 1-year incidence of respectively 31% (95% CI [21.4–41]) and 34.5% (95% CI [24.5–44.7]) in the ECP group, 21.4% (95% CI [11.8–33]) and 33.9% (95% CI [21.8–46.4]) in the Ruxolitinib group.

Overall, the most reported infections were bacteremia (36 patients), pneumonia (22 pts), upper respiratory tract infections (17 pts), CMV reactivation (14 pts) and skin infection (13 pts). Less frequent reported infections included urinary tract infections (5 pts), urinary tract infections (5 pts), invasive fungal infections (5 pts), clostridium difficile (2 pts) as well as various less frequent infections (9 pts).

In multivariate analysis adjusted on the grade of chronic GvHD and the type of steroid refractory, no significant difference was observed between patients who were treated with Ruxolitinib compared to patients treated with ECP regarding bacteraemia (HR 0.83, 95% CI [0.4–1.69], *p* = 0.60) or viremia (HR 0.98, 95% CI [0.53–1.8], *p* = 0.94).

### Patients receiving treatment with ECP and ruxolitinib

During the study period, we identified additional 55 alloSCT recipients with SR-cGVHD who were treated with a combination of ECP and ruxolitinib. There was variety in treatment durations and treatment sequences. These patients were not included in the current analyses. However, we observe that in some centers combination treatments of ECP and ruxolitinib were already in clinical use during the study period 2017-2019.

## Discussion

In the present retrospective study of SR-cGVHD treatment with ECP versus ruxolitinib, we extensively collected data using specifically designed data sheets (so called Med-C forms) and detected no statistically significant differences in major clinical parameters: overall response rate at day +180 as well as overall survival, progression-free survival, non-relapse mortality and relapse incidence. The clinical significance is limited by the retrospective study design and we need to be cautious with interpretation. Since there was no randomization certain factors leading to the choice of the one or the other therapy could bias the outcome. For example we do not have information why Ruxolitinib was used in some patients and ECP in others. We can’t conclude from this data that ECP is equally efficacious as compared to ruxolitinib in this indication. This question needs to be addressed in prospective studies on ECP in SR-cGVHD. However, our present results add more data to the already accumulating evidence on ECP as an effective treatment option in SR-cGVHD [5–11].

We found an overall response rate of ECP treatment in SR-cGVHD at day+180 of 45.7% in the ECP arm and 56.1% in the ruxolitinib arm, without statistically significant difference in multivariate analyses. Due to a variety in patient populations and also in SR-cGVHD definitions and treatment-response definition this is hard to compare the response rates observed in our study to results in the literature. However, overall our results in the ECP group are roughly in line with previously published evidence. In a meta-analysis of randomized controlled trials overall response data was extracted from five studies including 87 patients. These studies did not focus exclusively on SR-cGVHD. The pooled proportion of overall response rate of ECP during cGVHD was 64% with a high heterogeneity between studies [5]. A randomized study has tested either ECP and standard immunosuppressive therapy (n = 48) or standard therapy alone (n = 47) for treatment of cGVHD [7]. At week 12, 40% of the patients in the ECP arm had a complete or partial skin response, compared with 10% of the patients in the control arm (P = .002). Another prospective trial of ECP for cGVHD included 83 patients and found an overall response-rate of 62.3% [8]. The same is true for the response rates of ruxolitinib treatment in our study versus published evidence: it is not easily comparable but seems to be roughly in a similar range. We found 56.1% overall response rate of SR-cGVHD at day+180, whereas the seminal phase III trial resulted in 49.7% overall response rate at day week 24 [4]. Of note, in our retrospective analysis there were no predefined time points for response assessment leading to a selection of patients where response till day+180 was available. On top of this, it may lead to wrong assumptions to define response anytime till day+180 as opposed to exactly at day+180, because patients may initially respond and then lose the response later on. These are potentially major confounding factors adding to the limitations of this retrospective study.

An obvious possibility to increase response rates is to combine ECP with ruxolitinib treatments and we identified 55 alloSCT recipients with SR-cGVHD who were treated with such a combination during the study period 2017-19. Due to a variety in treatment durations and treatment sequences we decided not to include these cases in the current analyses and are therefore unable to provide new data on the combination of ECP and ruxolitinib versus monotherapy with only one of the substances. However, there is some published evidence as one retrospective study reported 23 patients receiving the combination of ruxolitinib and ECP as salvage therapy for SR-cGVHD [14]. The overall best response rate was 74% and the 24-months-survival was 75%. Newly diagnosed cytopenia occurred in 22% and CMV reactivation was observed in 26% of the patients. The authors concluded that the combination treatment is safe and has activity in a fraction of patients with SR-cGVHD, which needs validation in a prospective trial.

In the current study, we were specifiically interested in the patterns and frequencies of common infections complications in patiens with cGVHD. As expected, we found frequent infections in this high risk populations of SR-cGVHD patients. Of note, we did not find major differences regarding the type of infections in between ECP vs. ruxolitinib treated patients. The equally high infection frequency in the ECP and ruxolitinib arms are somehow surprising since there are theoretical benefits of ECP regarding the infection risk as compared with immunosuppressive therapies, such as ruxolitinib. ECP is believed to be rather immunomodulatory than exclusively immunosuppressive and supports a more anti-inflammatory cytokine profile as well as expansion of regulatory T-cells [6]. Our present results argue against a pronounced difference in infection risk between the two treatment modalities. Of note, roughly two thirds of patients have received additional immunosuppressive agents on top of the steroids as therapy of cGVHD prior to initiation of ECP or ruxolitinib, which also may have influenced the infection risk. The cumulative steroid burden that patients were exposed in both groups is also important. In our study, these data were only available at some time points. Therefore, we could not calculate the cumulative steroid dose. In addition, we are unable to give reliable information on immunosuppressive drugs co-administered with ruxolitinib or with ECP. There is the possibility that more immunosuppressive drugs were used in one or the other arm also influencing the infection risk. Overall, the conclusions regarding infection risk are limited by the fact that we are not able quantify the use of anti-infective prophylaxis in both arms, which may have impacted the incidence of infectious complications.

In conclusion we found no statistically significant differences in overall response rates and survival endpoints in patients with SR-cGVHD treated with ECP or ruxolitinib. The clinical significance is limited by the retrospective study design and the current data can’t replace prospective studies on ECP in SR-cGVHD. However, the present results contribute to the accumulating evidence on ECP as an effective treatment option in SR-cGVHD.

## Supplementary information

MED C Form

## Data Availability

Data is available on request to the corresponding author.
