# Correction: ECP versus ruxolitinib in steroid-refractory chronic GVHD – a retrospective study by the EBMT transplant complications working party

**DOI:** 10.1038/s41409-024-02435-8

**Published:** 2024-10-18

**Authors:** Olaf Penack, Christophe Peczynski, William Boreland, Jessica Lemaitre, H. Christian Reinhardt, Ksenia Afanasyeva, Daniele Avenoso, Tobias A. W. Holderried, Brian Thomas Kornblit, Eleni Gavriilaki, Carmen Martinez, Patrizia Chiusolo, Maria Caterina Mico, Elisabeth Daguenet, Stina Wichert, Hakan Ozdogu, Agnieszka Piekarska, Francesca Kinsella, Grzegorz W. Basak, Hélène Schoemans, Christian Koenecke, Ivan Moiseev, Zinaida Peric

**Affiliations:** 1 Medical Clinic, Department for Haematology, Oncology and Tumorimmunology, Charité Universitätsmedizin Berlin, Berlin, Germany; 2 EBMT Transplant Complications Working Party, Paris, France; 3 CHU de Saint-Etienne, Hematology, Saint-Etienne, France; 4 University Hospital, Essen, Germany; 5 RM Gorbacheva Research Institute, Pavlov University, St Petersburg, Russia; 6 Kings’ College Hospital, London, UK; 7 Department of Oncology, Hematology, Immuno-Oncology and Rheumatology, University Hospital Bonn, Bonn, Germany; 8 Rigshospitalet, Copenhagen, Denmark; 9 Papanicolaou G. Hospital, Thessaloniki, Greece; 10 Hematopoietic Stem Cell Unit, Hematology Department, ICMHO, Hospital Clínic of Barcelona, University of Barcelona, Barcelona, Spain; 11 Fondazione Policlinico Universitario A. Gemelli IRCCS-Università Cattolica del Sacro Cuore-Roma, Roma, Italy; 12 ASST Papa Giovanni XXIII, Bergamo, Italy; 13 Institut de Cancerologie Lucien Neuwirth, Saint-Étienne, France; 14 Skåne University Hospital in Lund, Lund, Sweden; 15 Department of Hematology, Baskent University Hospital, Adana, Türkiye; 16 Department of Hematology and Transplantology, University Clinical Center and Medical University of Gdansk, Gdańsk, Poland; 17 University Hospital Birmingham NHS Trust, Birmingham, UK; 18 Department of Hematology, Oncology and Internal Medicine, the Medical University of Warsaw, Warsaw, Poland; 19 Department of Hematology, University Hospitals Leuven, Leuven, Belgium; 20 Department of Public Health and Primary Care, ACCENT VV, KU Leuven - University of Leuven, Leuven, Belgium; 21 Department of Hematology, Hemostasis, Oncology and Stem Cell Transplantation, Hannover Medical School, Hannover, Germany; 22 Department of Haematology, University Hospital Centre Rijeka, Rijeka, Croatia

**Keywords:** Translational research, Stem-cell research

Correction to: *Bone Marrow Transplantation* 10.1038/s41409-023-02174-2 published online 06 January 2024

In this article, the author name Elisabeth Dagunet is incorrectly given. It should read Elisabeth Daguenet.

Also, the affiliation 3 EBMT Paris study office; Department of Haematology, Saint Antoine Hospital; INSERM UMR-S 938, Sorbonne University, Paris, France is given incorrectly. It should be CHU de Saint-Etienne, Hematology, Saint-Etienne. France.

The original article has been corrected.

