# Supplementary material for: ECP versus ruxolitinib in steroid-refractory chronic GVHD – a retrospective study by the EBMT transplant complications working party
Source: Bone Marrow Transplant. 2024 Jan 6;59(3):380–6. doi: 10.1038/s41409-023-02174-2 (PMC10920188; doi:10.1038/s41409-023-02174-2)
Supplement: Supplementary file 1 — MED C Form [file 41409_2023_2174_MOESM1_ESM.pdf]

Treatment of cGvHD (before SR status):

Steroid details:

Name of steroid	Start date	Initial dose *(mg/kg/day)*	Stop date
	_ _ _ _/_ _ /_ _		_ _ _ _/_ _ /_ _

Were other systemic drugs used to treat cGvHD (other than steroids): ☐ Yes ☐ No

If yes, please list drugs below:

Name of drug

Steroid Refractory cGvHD details:

SR cGvHD date of onset? _ _ _ _/_ _ /_ _

SR cGvHD type?
- ☐ *Steroid-refractory*
- ☐ *Steroid-dependent*
- ☐ *Steroid-intolerant*

cGvHD severity (at start of SR treatment)? _____________

cGvHD organ involved (at start of SR treatment):

	NIH score of cGvHD
Skin	☐ Score 0<br>☐ Score 1<br>☐ Score 2<br>☐ Score 3<br>☐ Not Scored

Liver	☐ Score 0<br>☐ Score 1<br>☐ Score 2<br>☐ Score 3<br>☐ Not Scored
Lower GI tract	☐ Score 0<br>☐ Score 1<br>☐ Score 2<br>☐ Score 3<br>☐ Not Scored
Upper GI tract	☐ Score 0<br>☐ Score 1<br>☐ Score 2<br>☐ Score 3<br>☐ Not Scored
Mouth	☐ Score 0<br>☐ Score 1<br>☐ Score 2<br>☐ Score 3<br>☐ Not Scored
Eyes	☐ Score 0<br>☐ Score 1<br>☐ Score 2<br>☐ Score 3<br>☐ Not Scored
Lung	☐ Score 0<br>☐ Score 1<br>☐ Score 2<br>☐ Score 3<br>☐ Not Scored
Other (specify):	☐ Score 0<br>☐ Score 1<br>☐ Score 2<br>☐ Score 3<br>☐ Not Scored

Treatment of the SR cGvHD

Start date of the SR treatment with ECP, Ruxolitinib or Ibrutinib - if more than one, whichever came first:

_ _ _ _/ _ _/ _ _
(yyyy/mm/dd)

ECP details:

Was ECP administered?	Start date *(yyyy/mm/dd)*	Initial schedule	**Schedule** *(after start of SR GvHD treatment with ECP)*						Reason for stopping treatment
			**At 90 days** *(or at closest possible date of evaluation)*	**At 180 days** *(or at closest possible date of evaluation)*	**At 360 days** *(or at closest possible date of evaluation)*	**At date of last assessment** (if > 390 days)	**Was ECP stopped for more than 6 weeks and restarted?**	**Is treatment ongoing?**	
☐ Yes<br>☐ No		**A** ☐ *3 treatments per week*<br>**B** ☐ *2 consecutive days per week*<br>**C** ☐ *2 consecutive days every two weeks*<br>**D** ☐ *1 day per week*<br>**E** ☐ *1 day every two weeks*<br>**F** ☐ *1 day per month*<br>**G** ☐ *2 consecutive days per month*<br>☐ *Other: _ _ _ _ _*	*Schedule:*<br>☐ **A**<br>☐ **B**<br>☐ **C**<br>☐ **D**<br>☐ **E**<br>☐ **F**<br>☐ **G**<br>☐ Other schedule:<br>_ _ _ _ _ _<br><br>*Date of evaluation:*<br>_ _ _ _/_ _/_ _	*Schedule:*<br>☐ **A**<br>☐ **B**<br>☐ **C**<br>☐ **D**<br>☐ **E**<br>☐ **F**<br>☐ **G**<br>☐ Other schedule:<br>_ _ _ _ _ _<br><br>*Date of evaluation:*<br>_ _ _ _/_ _/_ _	*Schedule:*<br>☐ **A**<br>☐ **B**<br>☐ **C**<br>☐ **D**<br>☐ **E**<br>☐ **F**<br>☐ **G**<br>☐ Other schedule:<br>_ _ _ _ _ _<br><br>*Date of evaluation:*<br>_ _ _ _/_ _/_ _	*Schedule:*<br>☐ **A**<br>☐ **B**<br>☐ **C**<br>☐ **D**<br>☐ **E**<br>☐ **F**<br>☐ **G**<br>☐ Other schedule:<br>_ _ _ _ _ _<br><br>*Date of evaluation:*<br>_ _ _ _/_ _/_ _	☐ Yes<br>☐ No<br><br>*Date stopped*<br>_ _ _ _/_ _/_ _<br>*Date restarted*<br>_ _ _ _/_ _/_ _	☐ Yes<br>☐ No<br><br>**If no:**<br>*Stop date*<br>_ _ _ _/_ _/_ _	☐ *Responded to treatment*<br>☐ *No response*<br>☐ *Stopped at patient request*<br>☐ *Adverse event*<br>☐ *Patient died*<br>☐ *Insufficient veneous access*<br>☐ *Not stated*<br>☐ *Other: _ _ _ _ _*

Ruxolitinib details:

**Was Ruxolitinib administered?**	**Start date** *(yyyy/mm/dd)*	**Initial dose**	**Dose** *(after start of SR GvHD treatment with Ruxolitinib)*						**Reason for stopping treatment**
			**At 90 days** *(or at closest possible date of evaluation)*	**At 180 days** *(or at closest possible date of evaluation)*	**At 360 days** *(or at closest possible date of evaluation)*	**At date of last assessment** (if > 390 days)	**Was Ruxolitinib stopped and restarted?**	**Is treatment ongoing?**	
☐ Yes<br>☐ No		☐ 10 mg twice a day<br>☐ 5 mg twice a day<br>☐ 5 mg once a day<br><br>Other: _ _ _	Dose: _ _ _ _ _ _<br>☐ mg once a day<br>☐ mg twice a day	Dose: _ _ _ _ _ _<br>☐ mg once a day<br>☐ mg twice a day	Dose: _ _ _ _ _ _<br>☐ mg once a day<br>☐ mg twice a day	Dose: _ _ _ _ _ _<br>☐ mg once a day<br>☐ mg twice a day	☐ Yes<br>☐ No	☐ Yes<br>☐ No	☐ *Responded to treatment*<br>☐ *No response*<br>☐ *Stopped at patient request*<br>☐ *Adverse event*<br>☐ *Patient died*<br>☐ *Not stated*<br>☐ *Other:* _ _ _ _
			Date of evaluation:<br>_ _ _ _/_ _/_ _	*Date of evaluation:*<br>_ _ _ _/_ _/_ _	*Date of evaluation:*<br>_ _ _ _/_ _/_ _	*Date of evaluation:*<br>_ _ _ _/_ _/_ _	*Date stopped:*<br>_ _ _ _/_ _/_ _<br><br>*Date restarted:*<br>_ _ _ _/_ _/_ _	**If no:**<br><br>*Stop date*<br>_ _ _ _/_ _/_ _	

Ibrutinib details:

**Was Ibrutinib administered?**	**Start date** *(yyyy/mm/dd)*	**Initial dose**	**Dose** *(after start of SR GvHD treatment with Ibrutinib)*						**Reason for stopping treatment**
			**At 90 days** *(or at closest possible date of evaluation)*	**At 180 days** *(or at closest possible date of evaluation)*	**At 360 days** *(or at closest possible date of evaluation)*	**At date of last assessment** (if > 390 days)	**Was Ibrutinib stopped and restarted?**	**Is treatment ongoing?**	
☐ Yes<br>☐ No		☐ 420 mg once a day<br>☐ 280 mg once a day<br>☐ 140 mg once a day<br><br>Other: _ _ _	Dose: _ _ _ _ _ _<br>*(mg once a day)*	Dose: _ _ _ _ _ _<br>*(mg once a day)*	Dose: _ _ _ _ _ _<br>*(mg once a day)*	Dose: _ _ _ _ _ _<br>*(mg once a day)*	☐ Yes<br>☐ No	☐ Yes<br>☐ No	☐ *Responded to treatment*<br>☐ *No response*<br>☐ *Stopped at patient request*<br>☐ *Adverse event*<br>☐ *Patient died*<br>☐ *Not stated*<br>☐ *Other:* _ _ _ _
			Date of evaluation:<br>_ _ _ _/_ _/_ _	*Date of evaluation:*<br>_ _ _ _/_ _/_ _	*Date of evaluation:*<br>_ _ _ _/_ _/_ _	*Date of evaluation:*<br>_ _ _ _/_ _/_ _	*Date stopped:*<br>_ _ _ _/_ _/_ _<br><br>*Date restarted:*<br>_ _ _ _/_ _/_ _	**If no:**<br><br>*Stop date*<br>_ _ _ _/_ _/_ _	

Steroid details:
(please note that time intervals are different for steroids than for ECP/Ruxolitinib/Ibrutinib)

Name of steroid	Start date *(yyyy/mm/dd)*	Dose at SR onset *(mg/kg/day)*	**Steroid dose** (*after start of SR GvHD treatment with ECP, Ruxolitinib or Ibrutinib - whichever comes first)*				Were steroids stopped and restarted?	Is treatment ongoing?
			**At 90 days** *(or at closest possible date of evaluation)*	**At 180 days** *(or at closest possible date of evaluation)*	**At 360 days** *(or at closest possible date of evaluation)*	**At date of last assessment** (if > 390 days)		
			Dose: _ _ _ _ _ _ *(mg/kg/day)*	Dose: _ _ _ _ _ _ *(mg/kg/day)*	Dose: _ _ _ _ _ _ *(mg/kg/day)*	Dose: _ _ _ _ _ _ *(mg/kg/day)*	☐ Yes ☐ No	☐ Yes ☐ No
			Date of evaluation: _ _ _ _/_ _ /_ _	*Date of evaluation:* _ _ _ _/_ _ /_ _	*Date of evaluation:* _ _ _ _/_ _ /_ _	*Date of evaluation:* _ _ _ _/_ _ /_ _	*Date stopped:* _ _ _ _/_ _ /_ _ *Date restarted:* _ _ _ _/_ _ /_ _	**If no:** *Stop date* _ _ _ _/_ _ /_ _

Were other systemic therapies used to treat SR cGvHD (other than steroids, Ruxolitinib or Ibrutinib): ☐ Yes ☐ No
This includes any treatments that were given before Ruxolitinib, Ibrutinib or ECP

If yes, please list drugs below:

Name of drug	Start date	Stop date
	_ _ _ _/_ _ /_ _	_ _ _ _/_ _ /_ _
	_ _ _ _/_ _ /_ _	_ _ _ _/_ _ /_ _
	_ _ _ _/_ _ /_ _	_ _ _ _/_ _ /_ _
	_ _ _ _/_ _ /_ _	_ _ _ _/_ _ /_ _
	_ _ _ _/_ _ /_ _	_ _ _ _/_ _ /_ _
	_ _ _ _/_ _ /_ _	_ _ _ _/_ _ /_ _

Response to the treatment of the SR cGvHD

Overall Response

Last recorded response to treatment: **Date of assessment:** _ _ _ _/_ _ /_ _

- ☐ *Complete response*
- ☐ *Partial response*

☐ *Stable disease*
☐ *Progressive disease*

Organ specific response (if available):

<table>
<tr><td rowspan="3">Organ</td><td colspan="5">NIH score of cGvHD (after start of SR GvHD treatment with ECP, Ruxolitinib or Ibrutinib - whichever comes first)</td><td rowspan="3">Date of resolution
(yyyy/mm/dd)</td></tr>
<tr><td>At 90 days (or at closest possible date of evaluation)</td><td>At 180 days (or at closest possible date of evaluation)</td><td>At 270 days (or at closest possible date of evaluation)</td><td>At 360 days (or at closest possible date of evaluation)</td><td>At date of last assessment
(if >390 days)</td></tr>
<tr><td>Date of evaluation:
_ _ _ _/_ _/_ _</td><td>Date of evaluation:
_ _ _ _/_ _/_ _</td><td>Date of evaluation:
_ _ _ _/_ _/_ _</td><td>Date of evaluation:
_ _ _ _/_ _/_ _</td><td>Date of evaluation:
_ _ _ _/_ _/_ _</td></tr>
<tr><td>Skin</td><td>☐ Score 0
☐ Score 1
☐ Score 2
☐ Score 3
☐ Not Scored</td><td>☐ Score 0
☐ Score 1
☐ Score 2
☐ Score 3
☐ Not Scored</td><td>☐ Score 0
☐ Score 1
☐ Score 2
☐ Score 3
☐ Not Scored</td><td>☐ Score 0
☐ Score 1
☐ Score 2
☐ Score 3
☐ Not Scored</td><td>☐ Score 0
☐ Score 1
☐ Score 2
☐ Score 3
☐ Not Scored</td><td>_ _ _ _/_ _/_ _</td></tr>
<tr><td>Liver</td><td>☐ Score 0
☐ Score 1
☐ Score 2
☐ Score 3
☐ Not Scored</td><td>☐ Score 0
☐ Score 1
☐ Score 2
☐ Score 3
☐ Not Scored</td><td>☐ Score 0
☐ Score 1
☐ Score 2
☐ Score 3
☐ Not Scored</td><td>☐ Score 0
☐ Score 1
☐ Score 2
☐ Score 3
☐ Not Scored</td><td>☐ Score 0
☐ Score 1
☐ Score 2
☐ Score 3
☐ Not Scored</td><td>_ _ _ _/_ _/_ _</td></tr>
<tr><td>Lower GI tract</td><td>☐ Score 0
☐ Score 1
☐ Score 2
☐ Score 3
☐ Not Scored</td><td>☐ Score 0
☐ Score 1
☐ Score 2
☐ Score 3
☐ Not Scored</td><td>☐ Score 0
☐ Score 1
☐ Score 2
☐ Score 3
☐ Not Scored</td><td>☐ Score 0
☐ Score 1
☐ Score 2
☐ Score 3
☐ Not Scored</td><td>☐ Score 0
☐ Score 1
☐ Score 2
☐ Score 3
☐ Not Scored</td><td>_ _ _ _/_ _/_ _</td></tr>
<tr><td>Upper GI tract</td><td>☐ Score 0
☐ Score 1
☐ Score 2
☐ Score 3
☐ Not Scored</td><td>☐ Score 0
☐ Score 1
☐ Score 2
☐ Score 3
☐ Not Scored</td><td>☐ Score 0
☐ Score 1
☐ Score 2
☐ Score 3
☐ Not Scored</td><td>☐ Score 0
☐ Score 1
☐ Score 2
☐ Score 3
☐ Not Scored</td><td>☐ Score 0
☐ Score 1
☐ Score 2
☐ Score 3
☐ Not Scored</td><td>_ _ _ _/_ _/_ _</td></tr>
<tr><td>Mouth</td><td>☐ Score 0
☐ Score 1
☐ Score 2
☐ Score 3
☐ Not Scored</td><td>☐ Score 0
☐ Score 1
☐ Score 2
☐ Score 3
☐ Not Scored</td><td>☐ Score 0
☐ Score 1
☐ Score 2
☐ Score 3
☐ Not Scored</td><td>☐ Score 0
☐ Score 1
☐ Score 2
☐ Score 3
☐ Not Scored</td><td>☐ Score 0
☐ Score 1
☐ Score 2
☐ Score 3
☐ Not Scored</td><td>_ _ _ _/_ _/_ _</td></tr>
<tr><td>Eyes</td><td>☐ Score 0
☐ Score 1
☐ Score 2</td><td>☐ Score 0
☐ Score 1
☐ Score 2</td><td>☐ Score 0
☐ Score 1
☐ Score 2</td><td>☐ Score 0
☐ Score 1
☐ Score 2</td><td>☐ Score 0
☐ Score 1
☐ Score 2</td><td>_ _ _ _/_ _/_ _</td></tr>
</table>

	☐ Score 3<br>☐ Not Scored	☐ Score 3<br>☐ Not Scored	☐ Score 3<br>☐ Not Scored	☐ Score 3<br>☐ Not Scored	☐ Score 3<br>☐ Not Scored	
Lung	☐ Score 0<br>☐ Score 1<br>☐ Score 2<br>☐ Score 3<br>☐ Not Scored	☐ Score 0<br>☐ Score 1<br>☐ Score 2<br>☐ Score 3<br>☐ Not Scored	☐ Score 0<br>☐ Score 1<br>☐ Score 2<br>☐ Score 3<br>☐ Not Scored	☐ Score 0<br>☐ Score 1<br>☐ Score 2<br>☐ Score 3<br>☐ Not Scored	☐ Score 0<br>☐ Score 1<br>☐ Score 2<br>☐ Score 3<br>☐ Not Scored	_ _ _ _/_ _/_ _
Other (specify):	☐ Score 0<br>☐ Score 1<br>☐ Score 2<br>☐ Score 3<br>☐ Not Scored	☐ Score 0<br>☐ Score 1<br>☐ Score 2<br>☐ Score 3<br>☐ Not Scored	☐ Score 0<br>☐ Score 1<br>☐ Score 2<br>☐ Score 3<br>☐ Not Scored	☐ Score 0<br>☐ Score 1<br>☐ Score 2<br>☐ Score 3<br>☐ Not Scored	☐ Score 0<br>☐ Score 1<br>☐ Score 2<br>☐ Score 3<br>☐ Not Scored	_ _ _ _/_ _/_ _
Overall severity	☐ No cGvHD<br>☐ Mild<br>☐ Moderate<br>☐ Severe<br>☐ Not Calculated	☐ No cGvHD<br>☐ Mild<br>☐ Moderate<br>☐ Severe<br>☐ Not Calculated	☐ No cGvHD<br>☐ Mild<br>☐ Moderate<br>☐ Severe<br>☐ Not Calculated	☐ No cGvHD<br>☐ Mild<br>☐ Moderate<br>☐ Severe<br>☐ Not Calculated	☐ No cGvHD<br>☐ Mild<br>☐ Moderate<br>☐ Severe<br>☐ Not Calculated	_ _ _ _/_ _/_ _
Overall response<br>*(if organ specific scoring is not available)*	☐ *Complete response*<br>☐ *Partial response*<br>☐ *Stable disease*<br>☐ *Progressive disease*<br>☐ Not Assessed	☐ *Complete response*<br>☐ *Partial response*<br>☐ *Stable disease*<br>☐ *Progressive disease*<br>☐ Not Assessed	☐ *Complete response*<br>☐ *Partial response*<br>☐ *Stable disease*<br>☐ *Progressive disease*<br>☐ Not Assessed	☐ *Complete response*<br>☐ *Partial response*<br>☐ *Stable disease*<br>☐ *Progressive disease*<br>☐ Not Assessed	☐ *Complete response*<br>☐ *Partial response*<br>☐ *Stable disease*<br>☐ *Progressive disease*<br>☐ Not Assessed	_ _ _ _/_ _/_ _

Infections, complications and comorbidities:

Were there any infections present during the treatment for SR GvHD or follow-up? ☐ Yes ☐ No

If yes, please complete the following table:

**Type**	**Pathogen involved** (please specify)	**Localisation**	**Start date** *(yyyy/mm/dd)*	**End date** *(yyyy/mm/dd)*	**Comments**
☐ Bacteraemia<br>☐ Fungemia<br>☐ Viremia<br>- CMV new or reactivation: _ _ _ _<br>☐ Parasites		☐ CNS<br>☐ Gut<br>☐ Lung<br>☐ Skin<br>☐ Other *(specify)*:<br>_ _ _ _ _ _ _ _ _ _ _ _			
☐ Bacteraemia<br>☐ Fungemia<br>☐ Viremia<br>- CMV new or reactivation: _ _ _ _<br>☐ Parasites		☐ CNS<br>☐ Gut<br>☐ Lung<br>☐ Skin<br>☐ Other *(specify)*:<br>_ _ _ _ _ _ _ _ _ _ _ _ _			
☐ Bacteraemia<br>☐ Fungemia<br>☐ Viremia<br>- CMV new or reactivation: _ _ _ _<br>☐ Parasites		☐ CNS<br>☐ Gut<br>☐ Lung<br>☐ Skin<br>☐ Other *(specify)*:<br>_ _ _ _ _ _ _ _ _ _ _ _ _			
☐ Bacteraemia<br>☐ Fungemia<br>☐ Viremia<br>- CMV new or reactivation: _ _ _ _<br>☐ Parasites		☐ CNS<br>☐ Gut<br>☐ Lung<br>☐ Skin<br>☐ Other *(specify)*:<br>_ _ _ _ _ _ _ _ _ _ _ _ _			
☐ Bacteraemia<br>☐ Fungemia<br>☐ Viremia<br>- CMV new or reactivation: _ _ _ _<br>☐ Parasites		☐ CNS<br>☐ Gut<br>☐ Lung<br>☐ Skin<br>☐ Other *(specify)*:<br>_ _ _ _ _ _ _ _ _ _ _ _ _			

☐ Bacteraemia<br>☐ Fungemia<br>☐ Viremia<br>- CMV new or reactivation: _ _ _ _<br>☐ Parasites		☐ CNS<br>☐ Gut<br>☐ Lung<br>☐ Skin<br>☐ Other *(specify):*<br>_ _ _ _ _ _ _ _ _ _ _ _ _			

Were there any complications present during the treatment for SR GvHD or the follow up? ☐ Yes ☐ No
If yes, please complete the following table:

**Type**	**Start date** *(yyyy/mm/dd)*	**End date** *(yyyy/mm/dd)*	**Comments**
Idiopathic pneumonia syndrome			
Haemorrhagic cystitis, non-infectious			
Aseptic bone necrosis			
ARDS, non-infectious			
Multiorgan failure, non-infectious			
Renal failure requiring dialysis			
Haemolytic anaemia due to blood group			
Transplant associated microangiopathy			
Hypertension			
Hepatitis / hepatic toxicity			
Cytopenia			
Thrombocytopenia			
Neutropenia			
Leucopenia			
Anaemia			
Thrombolytic / bleeding complications			
Hypotension			
Hypokalemia			
Leukoencephalopathy			
Pyrexia / fever			
Hypomagnesemia			
Renal impairment			
Tumor lysis syndrome			
Other complication *(please specify)*			

Other complication<br>*(please specify)*			
Other complication<br>*(please specify)*			
